# Status of Targeting MreB for the Development of Antibiotics

**DOI:** 10.3389/fchem.2019.00884

**Published:** 2020-01-10

**Authors:** Elvis Awuni

**Affiliations:** Department of Biochemistry, School of Biological Sciences, CANS, University of Cape Coast, Cape Coast, Ghana

**Keywords:** antibiotic targets, bacterial infections, druggable, therapeutics, prokaryotic actin homolog, MreB, cytoskeleton

## Abstract

Although many prospective antibiotic targets are known, bacterial infections and resistance to antibiotics remain a threat to public health partly because the druggable potentials of most of these targets have yet to be fully tapped for the development of a new generation of therapeutics. The prokaryotic actin homolog MreB is one of the important antibiotic targets that are yet to be significantly exploited. MreB is a bacterial cytoskeleton protein that has been widely studied and is associated with the determination of rod shape as well as important subcellular processes including cell division, chromosome segregation, cell wall morphogenesis, and cell polarity. Notwithstanding that MreB is vital and conserved in most rod-shaped bacteria, no approved antibiotics targeting it are presently available. Here, the status of targeting MreB for the development of antibiotics is concisely summarized. Expressly, the known therapeutic targets and inhibitors of MreB are presented, and the way forward in the search for a new generation of potent inhibitors of MreB briefly discussed.

## Introduction

The emergence of antibiotic-resistant bacterial strains (White et al., 2011; Ventola, 2015; Frieri et al., 2017; Li and Webster, 2018; Thorpe et al., 2018) has aggravated the challenges posed by bacterial infections to public health (Founou et al., 2017), resulting in the need to find new antibacterial agents. Nonetheless, the full druggable potentials of most bacterial pharmacological targets have yet to be tapped, and it is not surprising that few antibiotics have been released into the drug market in the past several years (Conly and Johnston, 2005). The bacterial actin-like MreB is a potential therapeutic target (Kruse et al., 2005; Vollmer, 2006; White and Gober, 2012) that has been widely studied but, unfortunately, has not been fully explored for the development of antibiotics. As an ATPase whose primary function involves coordinating bacterial cell wall biosynthesis (Figge et al., 2004), molecules that could inhibit MreB are prospective antibiotics. MreB is a promising drug target because it is conserved and essential in most rod-shaped bacteria (Varley and Stewart, 1992; Costa and Anton, 1993, 1999; Burger et al., 2000; Jones et al., 2001; Figge et al., 2004; Kruse et al., 2005; Slovak et al., 2005; Mazza et al., 2006; Robertson et al., 2007; Bean and Amann, 2008), except the few species that grow by tip extension (Margolin, 2009; Flärdh et al., 2012).

Notwithstanding that much is known about the structure and function of MreB and the possibility of targeting it with inhibitors (Kruse et al., 2005; Vollmer, 2006; White and Gober, 2012), no approved antibiotics against MreB are currently available. In this mini-review, the status of targeting MreB for the development of antibiotics is concisely summarized. Precisely, the known drug targets and inhibitors of MreB are presented, and the way forward in the quest to discover/develop a new generation of MreB inhibitors briefly discussed.

## Structure, Organization, and Function of MreB

**Figure 1:**
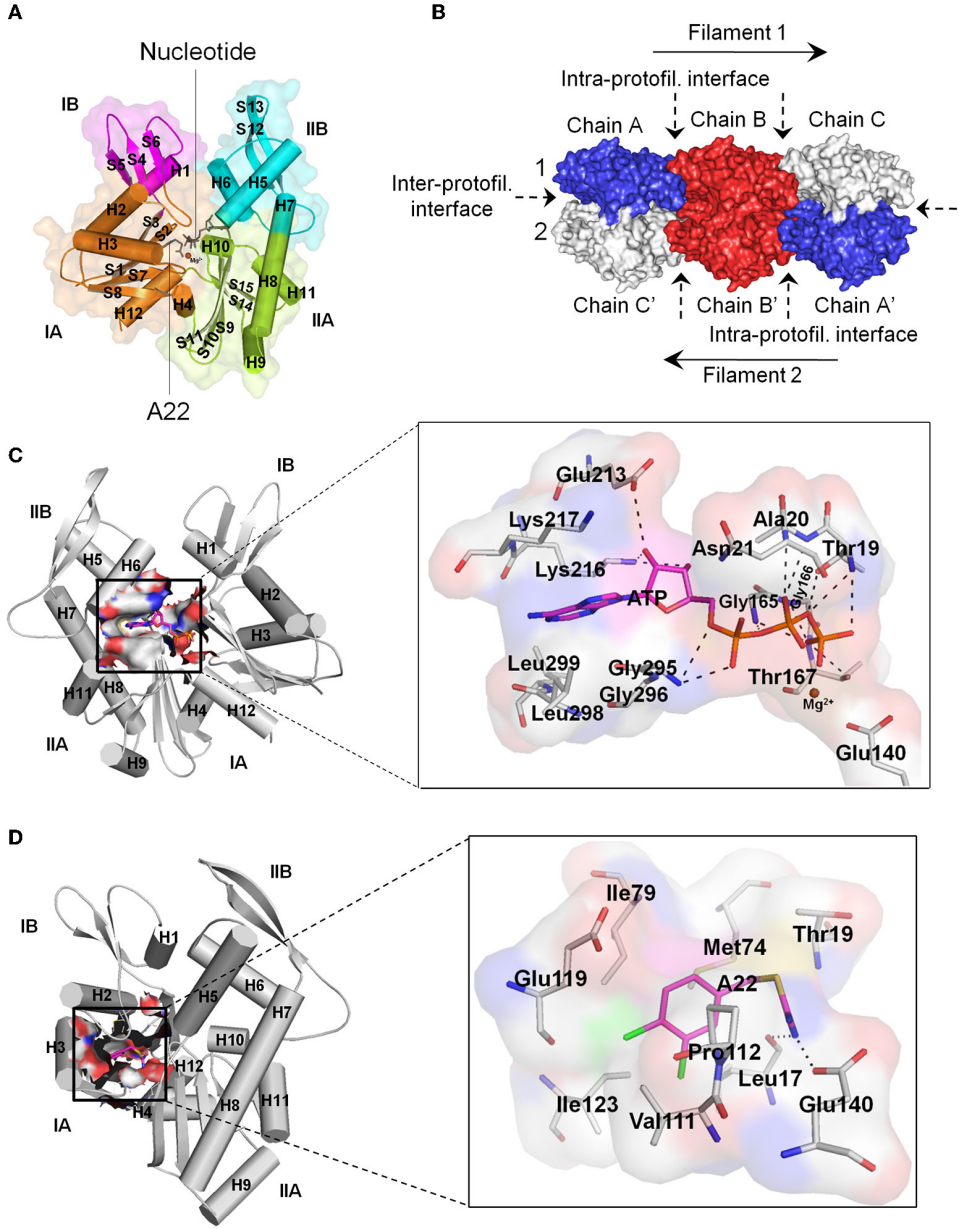
Structural features of MreB. **(A)** Monomeric MreB showing the subdomains IA, IB, IIA, and IIB as well as the nucleotide and A22 binding sites. Alpha helices and beta sheets are labeled as H and S respectively. This figure was prepared using the crystal structure of CcMreB with PDB ID: 4CZK. **(B)** Double protofilament MreB consisting of monomeric structures interacting at the intra- and inter-protofilament interfaces. This figure was prepared using the crystal structure of CcMreB with PDB ID: 4CZJ. **(C)** The ATP binding site of MreB showing the amino acid residues that make important interactions and contacts with ATP. The triphosphate group of ATP forms hydrogen bonds with Thr19, Ala20, Asn21, Gly165, Gly166, Thr167, and Gly295. The ribose sugar group of ATP forms hydrogen bonds with Glu213 and Lys216. The adenine ring, on the other hand, fits into a hydrophobic pocket created by the carbon atoms of the side-chains of Lys216, Lys217, Gly295, Gly296, Leu298, and Leu299. The Mg^2+^ atom stabilizes the γ-phosphate group of ATP and the catalytic Glu140 amino acid residue. The catalytic Glu140 localizes a catalytic water molecule through hydrogen bonding and acts as a base to facilitate the transfer of a proton from the water molecule to generate a nucleophile, which mounts a nucleophilic attack on the γ-phosphorous atom of ATP leading to ATP hydrolysis (Matte et al., 1998; Harrison and Schulten, 2012). **(D)** The A22/MP265 binding site of MreB showing the amino acid residues that make important interactions and contacts with A22. The isothiourea moiety of A22 forms hydrogen bonds with Leu17 and the catalytic Glu140 amino acid residues. On the other hand, the dichlorobenzyl group fits into a hydrophobic core created by the carbon atoms of Leu17, Met74, Ile79, Val111, Pro112, Glu119, and Ile123. Also, the pyrrolidine ring of Pro112 and the benzene ring of A22 are involved in a π-stacking interaction. For the ATP and A22/MP265 binding sites, carbon atoms of ATP and A22 are colored in magenta, and carbon atoms of amino acid residues are colored in gray. Nitrogen, oxygen, phosphorus, magnesium, sulfur, and chlorine atoms are colored blue, red, orange, brown, yellow, and green, respectively. Hydrogen bonds are shown as black dashes. **(C,D)** Prepared using the crystal structure of CcMreB with PDB ID: 4CZK. All images were prepared using the Pymol molecular visualization tool.

MreB is encoded by gene B of the mre cluster of genes and belongs to the actin/Hsp 70 superfamily. MreB is referred to as the prokaryotic actin by virtue of the similarity between its monomeric three-dimensional tertiary structure and that of eukaryotic actin (Van Den Ent et al., 2001), and its ability to form filamentous polymers *in vitro* (Van Den Ent et al., 2001; Esue et al., 2005, 2006) and *in vivo* (Jones et al., 2001; Srinivasan et al., 2007). Monomeric MreB consist of two main domains, I and II, which are divided into two alpha/beta subdomains to give rise to four subdomains namely IA, IB, IIA, and IIB, with a nucleotide binding site located within the interdomain cleft (Figure 1A; Van Den Ent et al., 2001). Crystal structures show that MreB monomers interact longitudinally at the intra-protofilament interfaces and laterally at the inter-protofilament interfaces to form double protofilaments (Figure 1B; Van Den Ent et al., 2001, 2014) that associate with the cell membrane (Salje et al., 2011; Schirner et al., 2015). Unlike the twisted and parallel strands of eukaryotic actin, the two strands of MreB are straight and antiparallel (Van Den Ent et al., 2014). MreB polymers are dynamic structures (Carballido-Lopez and Errington, 2003; Defeu Soufo and Graumann, 2004; Kim et al., 2006; Allard and Rutenberg, 2007; Biteen et al., 2008), and rotate around the length of the cell (Domínguez-Escobar et al., 2011; Garner et al., 2011; Van Teeffelen et al., 2011). It has been revealed that MreB assembles into discrete patches (Bendezú et al., 2009; Domínguez-Escobar et al., 2011) contrary to an earlier view that it organizes into helical filaments *in vivo* (Carballido-Lopez, 2006).

MreB has been associated with essential subcellular processes including cell wall biosynthesis and maintenance of cell shape (Doi et al., 1988; Jones et al., 2001), cell division (Wachi and Matsuhashi, 1989), chromosome segregation (Kruse et al., 2003; Soufo and Graumann, 2003; Gitai et al., 2005; Kruse and Gerdes, 2005), cell wall morphogenesis (Soufo and Graumann, 2005), and cell polarity (Gitai et al., 2004) in most rod-shaped bacteria. Mutations in the MreB gene transform rod-shaped bacterial cells to spherical ones indicating that MreB plays a role in determining the rod shape (Wachi et al., 1987). It has been shown that MreB is needed for the correct localization of MurG, which catalyzes the last step in peptidoglycan precursor synthesis, as well as the organization of other murein biosynthetic enzymes including MraY, MurB, MurC, MurE, and MurF (Varma and Young, 2009; White et al., 2010; Kawai et al., 2011; Typas et al., 2011; Schirner et al., 2015). The bacterial actin MreB is also involved in the localization of the gliding motility complexes in *Myxococcus xanthus* (Mauriello et al., 2010; Fu et al., 2018), the pilus-associated proteins in *Pseudomonas (P) aeruginosa* (Cowles and Gitai, 2010), and the autotransporter protein IcsA in *Shigella* for actin tail formation (Krokowski et al., 2019).

## Targets of MreB for Antibiotic Discovery and Development

Currently, the nucleotide binding site, the A22 binding pocket, and the inter-protofilament interface of MreB have been identified as potential targets for antibiotics. The nucleotide binding site is an important target for antibiotics development because nucleotide binding plays a crucial role in the structure and dynamics of MreB (Bean and Amann, 2008; Colavin et al., 2014; Van Den Ent et al., 2014; Awuni et al., 2016; Awuni and Mu, 2019). ATP induces the polymerization of MreB into filaments required for cell wall biosynthesis (Van Den Ent et al., 2001; Popp et al., 2010; Awuni and Mu, 2019). Interestingly, the polymerization of MreB induces ATP hydrolysis, which serves as a timing process to coordinate depolymerization (Esue et al., 2005; Bean and Amann, 2008; Popp et al., 2010; Gunning et al., 2015). Thus, ATP is required by MreB to function properly and any molecule that could compete with ATP for binding to the nucleotide binding pocket could be a bactericidal agent. Figure 1C shows the amino acid residues in the nucleotide binding site of *Caulobacter crescentus* MreB (CcMreB) (PDB ID: 4CZK) that establish important interactions and contacts with ATP. These amino acid residues include Thr19, Ala20, Asn21, Glu140, Gly165, Gly166, Thr167, Glu213, Lys216, Lys217, Gly295, Gly296, Leu298, and Leu299.

After cocrystal structures revealed the binding site of A22 in MreB (Van Den Ent et al., 2014), the A22 binding pocket has since been considered as a propitious target for the development of antiMreB agents. The A22 binding site is located adjacent to the nucleotide binding site (Figure 1A). The main function of this pocket has yet to be made fully clear but it has been suggested that it forms the channel through which P_i_ is released after ATP hydrolysis (Van Den Ent et al., 2014). Figure 1D illustrates the amino acid residues in the A22 binding site of CcMreB (PDB ID: 4CZK) that make important interactions and contacts with A22. The amino acid residues involved include Leu17, Thr19, Met74, Ile79, Val111, Pr0112, Glu119, Ile123, and Glu140. The inter-protofilament interface of MreB has also been suggested as a possible target for the development of antibiotics following a recent (Heller et al., 2017) observation that the protein inhibitor CbtA interacts with the dimerization helix (H3) and important amino acid residues on this interface.

## Inhibitors of MreB

### A22 and Its Analogs

**Figure 2:**
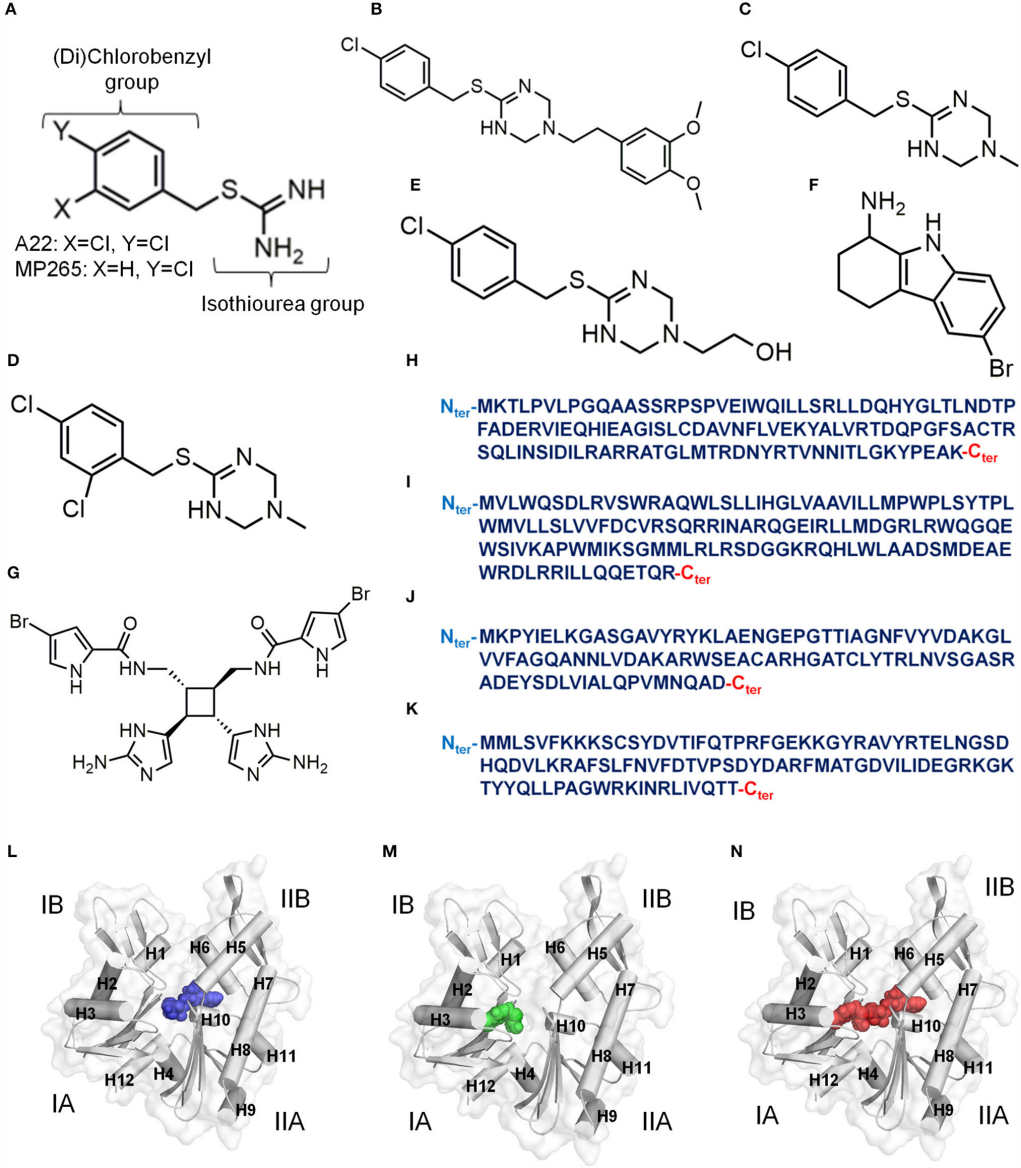
Inhibitors of MreB. **(A)** Chemical structure of A22/MP265. **(B)** Chemical structure of MAC13234. **(C)** Chemical structure of compound A. **(D)** Chemical structure of Compound 1. **(E)** Chemical structure of Compound 4. **(F)** Chemical structure of CBR-4830. **(G)** Chemical structure of Sceptrin. **(H)** Primary structure (124 amino acids, UniProt ID: P64524) of *Escherichia coli* CbtA. **(I)** Primary structure (135 amino acids, UniProt ID: Q46824) of *Escherichia coli* CptA. **(J)** Primary structure (100 amino acids) of *Caulobacter crescentus* MbiA (Yakhnina and Gitai, 2012). **(K)** Primary structure (104 amino acids, UniProt ID: O30472) of *Bacillus subtilis* YodL. **(L)** A model for an ATP-competitive inhibitor colored blue. **(M)** A model for a P_i_ channel blocker colored green. **(N)** A model for an ATP competitor and P_i_ channel blocker colored red. The four subdomains of MreB are indicated by IA, IB, IIA and IIB. Alpha helices in **(L–N)** are labeled as **H**.

A22 and its less-cytotoxic and much more water-soluble analog MP265 (Figure 2A) disrupt the rod shape of bacterial cells and induce coccoid shape (Iwai et al., 2002, 2004, 2007) by interacting with MreB (Bean et al., 2009). A22 was discovered in a study (Iwai et al., 2002) in which the anucleate cell blue assay (Wachi et al., 1999) was used to randomly screen a chemical library for inhibitors of chromosome partitioning in *Escherichia (E) coli*. MP265 was later discovered in a structure-activity relationship study of S-benzylisothiourea derivatives (Iwai et al., 2004, 2007). The binding modes and mechanisms of A22 and MP265 are similar but A22 is more effective against bacteria (Iwai et al., 2004, 2007; Van Den Ent et al., 2014). By targeting MreB, A22 interferes with the MreB-associated subcellular processes such as cell wall biosynthesis, cell division (Wachi and Matsuhashi, 1989), chromosome segregation (Kruse et al., 2003; Soufo and Graumann, 2003; Gitai et al., 2005; Kruse and Gerdes, 2005), cell wall morphogenesis (Soufo and Graumann, 2005), and cell polarity (Gitai et al., 2004) that are needed for the viability of most rod-shaped bacteria. A22 has micromolar binding affinity for MreB and interferes with its time course and extent of polymerization into filaments (Bean et al., 2009). By altering cell shape, A22 is also able to inhibit the motility, surface adhesion, and biofilm formation properties required for bacterial infections and antibiotic resistance (Bonez et al., 2017). A22 is more effective against Gram-negative bacteria than Gram-positive cells (Iwai et al., 2002, 2004, 2007; Noguchi et al., 2008; Nicholson et al., 2012; Carnell et al., 2018), probably due to variations in the amino acid sequence of the A22 binding pocket of the two strains (Bork et al., 1992; Jones et al., 2001; Van Den Ent et al., 2001). Although A22 has not yet been a useful therapeutic and is amenable to resistance following suppressor mutations (Shiomi et al., 2013), its low cytotoxic and genotoxic effects on human peripheral blood mononuclear cells (Bonez et al., 2016) makes it an important lead compound.

MAC13234 (Figure 2B) was discovered through a chemical genomics technique in *E. coli*, and was suggested to inhibit the LolA protein (Pathania et al., 2009). However, it was later found out that MAC13234 undergoes acid hydrolysis to generate MP265 (Barker et al., 2013), suggesting that it also targets MreB in an A22-like manner. A study (Yamachika et al., 2012) involving a whole-cell screening assay led to the discovery of compound A (Figure 2C), which showed anti-aeruginosa activity by targeting MreB. Like MAC13234, compound A is also believed to target MreB in the A22-like fashion by hydrolyzing to produce MP265. A more recent study (Buss et al., 2019) adopted a pathway-directed whole-cell screening technique to identify compounds 1 and 4 (Figures 2D,E) as active against *E. coli* by targeting MreB. Compounds 1 and 4 are also believed to inhibit MreB by hydrolyzing to produce A22 analogs.

### Mechanism of A22

The mechanism of A22 is yet to be clearly understood. A22 was thought to be an ATP-competitive inhibitor, and thus binds to the nucleotide binding site of MreB (Bean et al., 2009). However, X-ray crystallographic studies involving CcMreB, ATP, and A22 (Van Den Ent et al., 2014) have provided a contrary but convincing evidence that A22 and ATP bind independently to MreB at different and distinct sites (Figure 1A). By examining the crystal structures, Van Den Ent et al. (2014) proposed that A22 inhibits MreB by impeding ATP hydrolysis through interactions with the catalytic Glu140 amino acid residue (Figures 1C,D) and the γ-phosphate group of ATP, blocking the channel through which P_i_ is released after ATP hydrolysis, and disrupting double protofilament formation by displacing the main dimerization helix (H3) at the inter-protofilament interface. However, the idea that A22 inhibits ATP hydrolysis remains questionable given the observation that the MreB-ATP-A22 crystal complex has not been solved, in spite of the MreB-AMPPNP(a non-hydrolyzable ATP analog)-A22 complex being attainable. The notions that A22 blocks the release of P_i_ and impedes the formation of double protofilaments are, however, appealing for further investigations.

In a molecular dynamics simulations study (Awuni et al., 2016), it was observed that in the presence of ATP, A22 has the propensity to interact with the γ-phosphate group of ATP than the catalytic amino acid residue. The data therein also suggested that in both the ATP-bound and ATP-A22-bound states of MreB, water molecules enter the catalytic zone and are properly oriented to initiate the process of ATP hydrolysis. These observations suggest that A22 may not be an inhibitor of ATP hydrolysis as proposed earlier (Van Den Ent et al., 2014). The same study demonstrated further that the mechanism of A22 partly involves its ability to impede the release of P_i_ from the active site of MreB after ATP hydrolysis, leading to filament instability. A more recent similar study (Awuni and Mu, 2019) showed that A22 inhibits MreB in part by impeding an ATP-induced conformational change that it requires to polymerize into stable double protofilaments. It does appear that the mechanism of A22 is multidimensional and involves several effects on the structure and dynamics of MreB.

### Other Inhibitors

Although most of the known inhibitors of MreB are benzyl-isothiourea derivatives, some unique ones including CBR-4830, sceptrin, and the small proteins; CbtA, CptA, Gp0.6, MbiA, and YodL have been reported. CBR-4830 (Figure 2F) is an indole-class compound that was identified via a whole-cell antibacterial screen, and was shown to inhibit the growth of an efflux-compromised *P. aeruginosa* and induced coccoid shape (Robertson et al., 2007). It is believed that CBR-4830 inhibits MreB by competing with ATP for binding to the nucleotide binding pocket (Robertson et al., 2007). It has also been reported that CBR-4830 inhibits the growth of wild-type *E. coli* and induces spherical cells but may have significant off-target activity (Buss et al., 2019). Sceptrin (Figure 2G) is a natural product isolated from a marine sponge, *Agelas confera* (Walker et al., 1981; Cipres et al., 2010), and seems to have affinity for MreB (Rodríguez et al., 2008). Thus, sceptrin is suspected to have some anti-bacterial-cytoskeleton properties. CbtA (Figure 2H) is a toxin of *E. coli* origin that inhibits cell division and elongation by targeting FtsZ and MreB (Tan et al., 2011; Heller et al., 2017). A recent study (Heller et al., 2017) demonstrated that CbtA independently interacts directly with MreB at the inter-protofilament interface, and perhaps blocks the formation of double protofilaments. Interestingly, the protein antitoxin CbeA antagonizes the effects of CbtA and A22 by directly binding to MreB and FtsZ and enhancing the bundling of their filaments (Masuda et al., 2012a). Thus, the active component of CbeA could be optimized to produce an antibiotic as it could interfere with the polymerization-depolymerization dynamics of MreB. CptA (Figure 2I) is also an *E. coli* toxin that inhibits the polymerization of MreB and FtsZ, and its effect is neutralized by the antitoxin CptB (Masuda et al., 2012b). Gp0.6 (Molshanski-Mor et al., 2014), MbiA (Figure 2J) (Yakhnina and Gitai, 2012), and YodL (Figure 2K) (Duan et al., 2016) are also proteins that perturb cell shape by targeting MreB. Although the mechanisms of these inhibitors/modulators are not yet clear, they prove to be promising leads for the development of new antibiotics.

## Discussion and Future Directions

Much as advancements in science and technology have revolutionized the antibiotics discovery process, approved therapeutics against MreB are still not available. Targeting the ATP binding site of MreB for the development of antibiotics has not recorded any major successes partly because the development of ATP-competitive inhibitors is normally not so appealing to researchers as the inhibitors stand a higher chance of being non-specific as a consequence of the conserved nature of ATP binding sites among prokaryotes and eukaryotes (Škedelj et al., 2011). Furthermore, there are skepticisms about developing ATP-competitive inhibitors because to show *in vivo* activity, they must successfully compete with the high concentration of ATP in the bacterial cell (Gribble et al., 2000; Imamura et al., 2009). Nonetheless, there are successes made in finding ATP-competitive inhibitors against some bacterial targets (Lewis et al., 1996; Mochalkin et al., 2008, 2009; Silver, 2008). Thus, the ATP binding site of MreB remains a promising target for antibiotics development. Also, apart from A22 and its analogs for the A22 binding pocket, and CbtA for the inter-protofilament interface, no significant breakthroughs have been reported for these targets partly because the possibility of targeting them were recently (Van Den Ent et al., 2014; Heller et al., 2017) revealed.

The observations made in studies involving MreB-A22 interactions (Van Den Ent et al., 2014; Awuni et al., 2016; Awuni and Mu, 2019), suggest that the way forward to find potent antibiotics against MreB is to develop molecules that could effectively do one, a combination, or all of the following: (i) competitively exclude ATP from its binding site in MreB by occupying the nucleotide binding site as shown in Figure 2L, (ii) inhibit ATP hydrolysis by MreB by interacting strongly with the catalytic amino acid residue and the γ-phosphate group of ATP, and displacing water molecules from the catalytic zone by occupying the A22 binding pocket and the catalytic zone as illustrated in Figure 2M, (iii) obstruct the release of P_i_ from the active site of MreB by blocking the supposedly P_i_ channel as shown in Figure 2M, (iv) occupy the ATP and the A22 binding sites as illustrated in Figure 2N, and (v) induce a conformational change in MreB that will not favor its assembly into protofilaments. High throughput screening, virtual screening and other drug discovery strategies could be applied to identify such inhibitors. However, with the binding sites known and characterized, rational drug design approaches could be useful.

Even though small organic molecules remain relevant in the search for a new generation of antibiotics targeting MreB, the exploration of the prospects of antimicrobial peptides (AMPs) is attractive especially when there are reports of some protein inhibitors and modulators of MreB (Tan et al., 2011; Masuda et al., 2012a,b; Yakhnina and Gitai, 2012; Molshanski-Mor et al., 2014; Duan et al., 2016; Heller et al., 2017). AMPs are typically 12–50 amino acids long. However, the lengths of most of the MreB-targeting protein inhibitors and modulators mentioned herein are >50 amino acid residues, and thus they could serve as sources for developing optimized AMPs through the use of appropriate strategies to create peptide fragments for the determination of the minimum effective peptide lengths for inhibition. Interestingly, CbtA is reported to inhibit bacteria by targeting MreB and FtsZ proteins (Tan et al., 2011; Masuda et al., 2012b). FtsZ is the prokaryotic homolog of the eukaryotic tublin, and has been identified as a potential target for the development of antibiotics following the critical role it plays in bacterial cell division (Erickson et al., 2010; Guan et al., 2018). By targeting MreB and FtsZ, CbtA could represent a good lead for the development of a new generation of antibacterial agents to combat drug resistance. AMPs have the advantage, over organic small molecule drugs, of producing non-toxic byproducts (Loffet, 2002) and usually exhibit multiple mechanisms making the development of resistance toward them by microbes rare. Even though AMPs are often disadvantaged by their weak ability to traverse the plasma membrane to reach their intended targets, attempts by researchers to develop lipidic and polymeric nanocarriers to deliver peptides (Santalices et al., 2017) make AMPs development promising.

## Author Contributions

The author confirms being the sole contributor of this work and has approved it for publication.

### Conflict of Interest

The author declares that the research was conducted in the absence of any commercial or financial relationships that could be construed as a potential conflict of interest.
